# Novel Pathways for Targeting Tumor Angiogenesis in Metastatic Breast Cancer

**DOI:** 10.3389/fonc.2021.772305

**Published:** 2021-12-03

**Authors:** Jordan A. Harry, Mark L. Ormiston

**Affiliations:** ^1^ Department of Medicine, Queen’s University, Kingston, ON, Canada; ^2^ Department of Biomedical and Molecular Sciences, Queen’s University, Kingston, ON, Canada; ^3^ Department of Surgery, Queen’s University, Kingston, ON, Canada

**Keywords:** angiogenesis, breast cancer, vascular endothelial growth factor, bone morphogenetic protein 9, notch signaling

## Abstract

Breast cancer is the most common cancer affecting women and is the second leading cause of cancer related death worldwide. Angiogenesis, the process of new blood vessel development from pre-existing vasculature, has been implicated in the growth, progression, and metastasis of cancer. Tumor angiogenesis has been explored as a key therapeutic target for decades, as the blockade of this process holds the potential to reduce the oxygen and nutrient supplies that are required for tumor growth. However, many existing anti-angiogenic approaches, such as those targeting Vascular Endothelial Growth Factor, Notch, and Angiopoietin signaling, have been associated with severe side-effects, limited survival advantage, and enhanced cancer regrowth rates. To address these setbacks, alternative pathways involved in the regulation of tumor angiogenesis are being explored, including those involving Bone Morphogenetic Protein-9 signaling, the Sonic Hedgehog pathway, Cyclooxygenase-2, p38-mitogen-activated protein kinase, and Chemokine Ligand 18. This review article will introduce the concept of tumor angiogenesis in the context of breast cancer, followed by an overview of current anti-angiogenic therapies, associated resistance mechanisms and novel therapeutic targets.

## Introduction

Breast cancer is the most common invasive cancer affecting women. Despite an array of new therapies, diagnostic advances, and increased screening, it remains the second leading cause of cancer related death, emphasizing the critical need for new treatment options (1). The high mortality rates associated with breast cancer are due to distant metastasis from the primary breast tumor to the bones, liver, lungs, or brain (1–3). Angiogenesis, the process of new blood vessel formation from the pre-existing vasculature, is a hallmark of cancer and has been implicated in the growth, progression, and metastasis of breast cancer (4, 5). Beyond enhancing the supply of oxygen and nutrients to the tumor, angiogenesis also contributes to its metastatic potential. The fragile, tortuous, permeable and hypermalleable nature of the newly formed blood vessels allows cancer cells to enter the vasculature and travel to other tissues in the body (6, 7). These malformed vessels also contribute to a hypoxic and acidic tumor microenvironment that favours the selection of more aggressive cancer cells (8).

Initial therapeutic approaches targeting the tumor vasculature were primarily aimed at inhibiting angiogenesis, based on the mindset that preventing capillary network formation would slow tumor growth and reduce metastasis (9). However, these anti-angiogenic therapies have been limited by drug resistance and side effects, including venous thromboembolic complications, hypertension, and hemorrhaging (10–12). By reducing the blood supply to tumors, these approaches also limit the ability for chemotherapies and immune cells to infiltrate these tissues (10–12). As an alternative, therapeutic approaches based on vascular normalization have been explored, with the goal of improving tissue perfusion to provide a functional vasculature for the delivery of intravenous radiotherapy and chemotherapy (13, 14). These contrasting approaches illustrate a critical point of debate in breast cancer research; should tumor vessel formation be inhibited to prevent oxygen and nutrients from reaching primary tumors, or should the vessels be normalized to reduce metastatic spread and improve pathways for therapeutic delivery?

This review will assess the current state of both anti-angiogenic and vascular normalization strategies for the treatment of breast cancer. The various mechanisms that tumors use to develop and maintain their blood supply will be summarized, followed by a review of the pre-clinical and clinical outcomes for conventional anti-angiogenic therapies targeting well-established players in angiogenic signaling, such as Vascular Endothelial Growth Factor (VEGF), Notch signaling and the Angiopoietins (Ang). Emerging avenues for modulating tumor vascularization will also be discussed, including approaches targeting bone morphogenetic protein-9 (BMP9) signaling, Sonic Hedgehog Pathway (Shh) inhibitors, Cyclooxygenase-2 (COX-2) inhibitors, p38-mitogen-activated protein kinase (p38-MAPK) pathway inactivation and Chemokine Ligand 18 (CCL18) inhibition.

## Mechanisms of Tumor Vascularization

### Sprouting Angiogenesis

**Figure 1:**
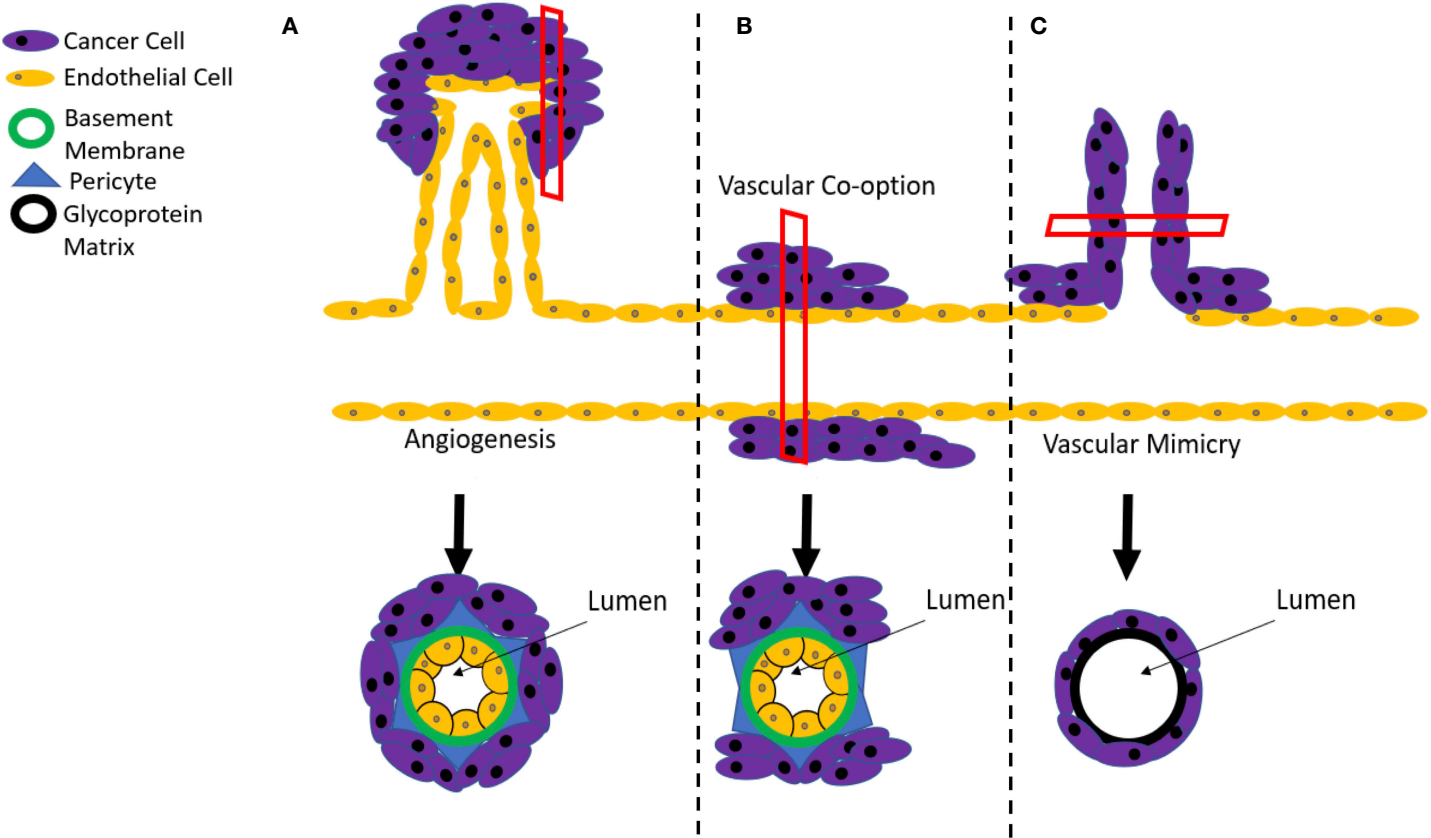
Mechanisms of tumor angiogenesis. **(A)** Tumor vascularization typically occurs by sprouting angiogenesis, involving the generation of new vessels *via* sprouting from the pre-existing circulation. However, resistance mechanisms to anti-angiogenic therapies can include **(B)** vascular co-option, where the tumor grows along existing vessels or **(C)** vascular mimicry where cancer stem cells create a *de novo* vasculature.

Sprouting angiogenesis is a primary source of new vascular growth in tumors (15). This process, which involves the growth of new capillary blood vessels from the pre-existing vasculature (**Figure 1A**), is highly regulated and occurs in healthy tissues through a fine balance of angiogenic and angiostatic cues (16). In tumors, changes in physiological stimuli, such as hypoxia (17), disrupt this balance and shift the process towards a pathogenic pro-angiogenic state (16). Sprouting angiogenesis is initiated by a process of growth factor-mediated endothelial cell activation, proliferation (5), loss of cell-cell junctions and decreased endothelial monolayer integrity (18). While this occurs, proteases degrade the extracellular matrix and basement membrane surrounding endothelial cells, which allows for their migration (5). Migrating endothelial cells are polarized into tip and stalk cell phenotypes, contribute to lumen formation, and eventually the establishment of immature blood vessels (5). In physiological angiogenesis, these immature vessels are then stabilized through angiostatic signals, resulting in tightened cell-cell junctions, the recruitment of mural cells and restoration of the basement membrane (19).

### The Tumor Microenvironment

In tumors, pathological angiogenic sprouting is perpetuated by a local microenvironment, composed of proliferating tumor cells, blood vessels, specialized immune cells, and inflammatory cytokines (20, 21). The increased expression of angiogenic factors, inflammation, and hypoxia in the tumor microenvironment is thought to contribute to the reduced efficacy of current anti-angiogenic therapies (22, 23). As tumors grow, high levels of oxygen are consumed. This, alongside reduced nutrients and the accumulation of metabolic wastes, creates a hypoxic and acidic microenvironment that persistently activates pro-angiogenic pathways, reduces the stabilization of vessels and promotes the formation of a leaky, disorganized vascular network (24, 25). The release of mitochondrial reactive oxygen species (ROS) by tumor cells and other cellular components of the microenvironment also helps to stabilize hypoxia-inducible factor (HIF) subunits when (26–28), further promoting the expression of VEGF and upregulating angiogenesis (29, 30).

Beyond the direct actions of VEGF on the sprouting endothelium, VEGF can also serve to promote an angiogenic and immunosuppressive microenvironment through the inhibition of dendritic cell maturation and the promotion of tumor associated macrophage (TAM) recruitment and polarization in the presence of Th2 cytokines like IL-4 and IL-10 (31–33). TAMs are a major contributor to the tumor microenvironment. Their production of growth factors, chemokines and proteolytic enzymes actively promote tumor angiogenesis, while also suppressing the antitumor immune response (34–36). The recruitment and polarization of TAMs from bone marrow or tissue-derived macrophages is driven by local factors, including chemokines like CCL2 and cytokines like IL-6, as well as VEGF and hypoxia (37–39).

### Vessel Co-Option

In addition to relying on sprouting angiogenesis, tumors can also be sustained through a process of vessel co-option (**Figure 1B**). This process involves solid tumors originating close to, and growing along, previously existing vessels (40). Vessel co-option is often distinguished from angiogenesis at the histopathological level, where tumors that achieve blood supply *via* co-option have a well-organized vascular network that resembles normal tissue and differs from the disorganized network that is produced by pathological sprouting angiogenesis (41). It is believed that breast cancer metastases to the brain, liver and lungs are sustained through this process, which consequently contributes to their resistance to conventional anti-angiogenic therapies (42–45). Although the process of vessel co-option has been linked to both innate and acquired resistance mechanisms to anti-angiogenic therapies (46), an increase in vessel co-option tends to proceed after angiogenic inhibition rather than precede it, indicating that it is more likely to be an acquired, rather than innate feature of the tumor (42).

### Vascular Mimicry

Vascular mimicry involves the development of a vascular-like structures within tumors that contain no endothelial cells and are instead composed of a basement membrane surrounded by cancer cells (**Figure 1C**) (47–52). This process has been attributed to the actions of a subset of cancer stem cells that possess a high degree of differentiation plasticity and can acquire a range of endothelial markers, including cluster of differentiation 31 (CD31), VEGF receptor-2 (VEGFR2), Tie2, ephrin A2, and VE-Cadherin (53, 54). Importantly, these markers enable the incorporation of mimicked vascular structures into endothelial-based vessels at the boundaries between tumors and normal tissue (55). A blood supply model for melanoma developed by Zhang and colleagues indicated that vascular mimicry is the dominant blood supply in early tumor growth, followed by the development of conventional endothelial-based vessels to support the later stages of tumor expansion (56). In addition to supporting growth, vascular mimicry also serves to increase the proportion of tumor cells that are located adjacent to blood flow, enhancing their likelihood of infiltrating the bloodstream and metastasizing to distant sites in the body (57). As a consequence, the occurrence of vascular mimicry is linked with distant metastases, poor overall survival, and local cancer relapse (58).

## Conventional Anti-Angiogenic Therapies

Several therapies targeting conventional angiogenic mediators, including the VEGF, Notch and Ang pathways, have been explored in both a pre-clinical and clinical setting, as summarized in **Table 1**.

**Table 1:** Summary of Available Therapies with the Ability to Target Tumor Angiogenesis in Breast Cancer.

Therapies	Mechanism of Action	Associated Trials	Outcomes
VEGF			
Bevacizumab	Monoclonal antibody for VEGF.	Pre-Clinical	small animal models demonstrated a reduction in tumor size and vascular permeability (59–61).
		Phase-II Clinical	linked to serious toxicities, including venous thromboembolic complications, hypertension, and hemorrhaging (11).
		Phase-III Clinical	No improvement in overall survival (62–65). A subset patients exhibited reduced metastatic disease progression, and minor improvements in PFS relative to controls (62–65).
Notch			
Demcizumab	Antibody targeting DLL4.	Phase-2 Clinical	Trial terminated as it failed to meet primary endpoint of overall response rate (66). Associated with cumulative CV toxicity (66).
Nirogacestat	Reversible, non-competitive GSI that selectively blocks Notch signaling.	Phase-1 Clinical	Small sample sizes and inconclusive findings in AMBC (67).
		Phase-3 Clinical	In recruitment phase (68).
Ang			
Trabananib	Peptide inhibitor that neutralizes the interactions of Ang1 and Ang2 with the Tie2 receptor.	Phase-2 Clinical	Underway for Her-2 positive breast cancer (69).
Ang2-VEGF CrossMab	Double specific anti-body against Ang2 and VEGF.	Pre-Clinical	Results in complete tumor regression in various tumor xenograft models, as well as a reduction in metastasis and angiogenesis (70).
		Phase-2	No improvement in PFS (71). Associated with gastrointestinal perforations, hypertension, and peripheral edema (71).

### VEGF Pathway Inhibitors

In humans and other mammals, the VEGF family of growth factors includes VEGF-A, VEGF-B, VEGF-C, VEGF-D and Placental Growth Factor (PlGF) (72). While some members of this family, such as VEGF-C and VEGF-D, act primarily on the lymphatic endothelium, VEGF-A, referred to hereafter as VEGF, is the most potent direct-acting protein involved in angiogenesis (73). As such, the VEGF pathway was among the first and most broadly interrogated targets for the development of anti-angiogenic therapeutics (73). The production and release of VEGF is stimulated by a variety of factors, with the most notable being tissue hypoxia (74, 75). VEGF initiates vascular permeability, as well as endothelial cell proliferation and migration (76), through the activation of multiple tyrosine kinase receptors, including VEGFR1 and VEGFR2, with the majority of angiogenic responses being mediated by VEGFR2 activation (77).

Bevacizumab, a monoclonal antibody for VEGF, is the main anti-angiogenic agent for use in breast cancer (78). It works by preventing the interaction of VEGF with its receptors, as well as through the neutralization of VEGF release from cancer cells (78). Bevacizumab is not approved as a monotherapy. However, it is approved to be given alongside chemotherapy for advanced or metastatic breast cancer (AMBC) (79, 80). In addition to the prevention of sprouting angiogenesis, Bevacizumab can also promote tumor vessel normalization by reducing vascular permeability and promoting the recruitment of pericytes that support the endothelium (81). While numerous pre-clinical studies in small animal models demonstrated a reduction in tumor size and vascular permeability with Bevacizumab (59–61), similar results have not been achieved in human trials.

In patients with AMBC, Bevacizumab did not improve disease related symptoms or overall survival (65). However, a subset of patients who received Bevacizumab did exhibit reduced metastatic disease progression, which resulted in a minor improvement in progression free survival (PFS) relative to controls (62–65). However, no improvement in overall survival has been reported (62–65). Part of Bevacizumab’s lack of efficacy may be attributable to resistance mechanisms like vessel co-option and vascular mimicry. In patients with colorectal cancer, the process of vessel co-option has been linked to poor responses to anti-angiogenic therapies like Bevacizumab (42). The trans-differentiated endothelial-like cancer cells that drive vascular mimicry have also shown limited sensitivity to anti-angiogenic therapeutics (55, 82), with tumors that are resistant to these therapies exhibiting higher levels of vascular mimicry (83).

In addition to this lack of efficacy, long term trials with Bevacizumab concluded that excessive neutralization of VEGF with high doses or prolonged exposure caused vascular regression and promoted hypoxia (84, 85), driving the selection of more invasive cancer cells that can contribute to tumor resistance and increased metastatic potential (84). Prolonged exposure to Bevacizumab did not just disrupt tumor vasculature, but also upregulated multiple angiogenic factors, including PlGF and stromal derived factor-1 (SDF-1), in addition to causing increased cancer cell migration and enhancing metastatic potential. Treatment with Bevacizumab also increased cancer regrowth rates after treatment cessation, which has been attributed to elevated tumor hypoxia levels and the upregulation of compensatory angiogenic pathways (86, 87).

Bevacizumab has also been linked to serious toxicities. Patients with colorectal cancer treated with Bevacizumab had >33% risk of developing any type of thrombosis (88). A meta-analysis of patients receiving Bevacizumab also identified a significantly increased risk of hypertension in this group (89). Hemorrhaging is also a widely reported side effect of Bevacizumab therapy, although the mechanisms underlying this and other side effects are not fully understood (11). The United States Food and Drug Administration (FDA) initially approved the use of Bevacizumab as a monotherapy for AMBC. However, this indication was later revoked due to considerable side effects, no convincing evidence of clinical benefit, and a lack of effective biomarkers to determine treatment response (90). Bevacizumab is still available on the market for prescription. However, its use in cancer is not approved by the FDA (91).

### Notch Signaling Pathway

The Notch signaling pathway plays a vital role in sprouting angiogenesis through the regulation endothelial cell proliferation, VEGF receptor expression and the commitment of sprouting endothelial cells to stalk or tip cell fates (92). As a consequence, there have been over 70 clinical trials therapeutically targeting Notch signaling in tumor angiogenesis (68). In canonical Notch signaling, Notch transmembrane receptors interact with Notch ligands, such as Delta-like 4 (DLL4) and Jagged-1 (JAG1), causing them to cleave and release their intracellular domain (93). In angiogenesis, DLL4 is induced by VEGF as a negative feedback regulator that induces the expression of *Hes/Hey* genes in stalk cells (94). These transcriptional regulators subsequently downregulate the expression of VEGFR2, making the cells less responsive to VEGF (95) and promoting the formation of a mature vascular network through the suppression of overexuberant angiogenic sprouting (96). Conversely, JAG1 alters the balance between DLL4-Notch and VEGFRs by antagonizing DLL4-mediated Notch activation. This reduction allows tip and stalk cells to change positions, resulting in a dense and tortuous vascular network (97). JAG1 also induces the expression of VEGF-receptor 3 (VEGFR3) (97), which influences the expression of both pro- and anti-angiogenic factors *via* the regulation of protein kinase B (AKT) signalling (98, 99).

In cancer cells, the balance of Notch signaling is pathogenically shifted so that the angiogenic actions of JAG1 dominate over the angiostatic effects of Notch-DLL4 (97). Despite this shift, Notch-DLL4 signalling has been therapeutically targeted using various inhibitory strategies, such as anti-DLL4 antibodies, DNA vaccinations, and Notch signaling inhibitors (100–102). Although blocking a pathway that is known to serve as an angiogenic “off switch” seems counterintuitive, agents targeting DLL4 have been shown to reduce tumor growth *in vivo* by promoting disorganized and non-productive endothelial sprouting and poor tumor perfusion (103). An antibody targeting DLL4, Demcizumab, was one of the first treatments to selectively target the Notch pathway in clinical trials (104). Demcizumab is a humanized monoclonal antibody for DLL4 that functions by blocking Notch receptor binding (104). Unfortunately, the phase 2 clinical trial for Demcizumab in pancreatic cancer was discontinued, as clinical outcomes were superior in the placebo arm over the treatment arm (66, 105). In addition to this failure to meet the primary endpoint of overall response rate, Demcizumab was also associated with cumulative cardiovascular (CV) toxicity (66), further hampering the translational potential of this approach.

Notch signaling in cancer has also been targeted using gamma secretase inhibitors (GSIs), which block the protease complex that cleaves Notch transmembrane domains (67). Despite their initial promise, GSIs have been linked to significant gastrointestinal toxicity, caused by the inhibition of Notch signaling in the stem-cell progenitor compartment of intestinal crypts (106, 107). As GSIs also cleave membrane proteins in various other signaling pathways, their non-specific inhibition can result in systemic toxicity (40, 108). Nirogacestat, the first Notch GSI to begin phase 3 clinical trials, is a reversible, non-competitive GSI that selectively blocks the Notch signaling pathway (67). A recent clinical trial assessing the impact of Nirogacestat on AMBC had small sample sizes and inconclusive findings (67). The ongoing phase 3 trial is currently in its recruitment phase (68), but should provide definitive insights into the ultimate potential of this approach.

Alternative strategies, such as the selective upregulation of DLL4 signaling with a soluble DLL4-Fc have also been explored to treat tumor angiogenesis (109). Pre-clinical studies based on this approach have demonstrated increased tumor vessel density and mural cell recruitment, along with decreasing tumor size (110), indicating potential merit in future clinical studies.

### Ang Pathway Inhibitors

Ang1 and Ang2 regulate angiogenesis and vascular remodeling through their binding to the endothelial receptor tyrosine kinase, Tie2 (111). The activation of Tie2 is associated with vascular stabilization, *via* increased pericyte coverage and decreased blood vessel permeability (112, 113). Consequently, Tie2 activation has been linked to increased vessel diameter, vascular density, and perfusion within tumors (112, 113). Although both Ang1 and Ang2 bind Tie2 with similar affinity, only Ang1 promotes vascular development and maturation *via* robust receptor activation. In contrast, Ang2 is viewed as an Ang1 antagonist (114) that only weakly activates Tie2 (112, 113). In the presence of VEGF, Ang2 promotes vascular sprouting and destabilizes blood vessels through reduced endothelial-pericyte interactions (114). In contrast, when VEGF is absent, Ang2 accelerates blood vessel regression and promotes endothelial cell death (115).

Ang2 is highly expressed in breast cancer and has been linked to tumor angiogenesis in AMBC (116), making it an exciting therapeutic target (113). Therapies targeting Ang signaling in tumor angiogenesis include Trebananib, a peptide inhibitor that can neutralize the interactions of Ang1 and Ang2 with the Tie2 receptor (69). Trebananib has been used as a combination therapy for Her2-negative breast cancer and is currently in phase 2 clinical trials for ovarian cancer and Her2-positive breast cancer (69). This combination treatment has shown promising anti-tumor activity, especially in solid tumors (117). Ang2-VEGF CrossMab is another therapy targeting angiogenesis through the Ang2 pathway. This is a double specific antibody against both Ang2 and VEGF that causes complete tumor regression in various tumor xenograft models, as well as a reduction in metastasis and angiogenesis (70). Despite pre-clinical success, clinical trials of Ang2-VEGF CrossMab did not show improved PFS and were instead associated with increased anti-angiogenic toxicity, such as gastrointestinal perforations, hypertension, and peripheral edema (71). Overall, there is a promising amount of research targeting tumor angiogenesis through the Ang pathway. However, to date, these therapies have had limited success, and have only been used in combination with other chemotherapies, or anti-angiogenic agents.

## The Need for Alternative Pathways for Breast Cancer Therapeutics

As detailed above, there is a significant need for novel targets and approaches that improve upon the toxicities, limited survival advantage and enhanced cancer regrowth rates associated with current anti-angiogenic therapies (118). In addition to conventional angiogenic mediators like VEGF, Notch and Ang(s), the last decade has seen the rise of multiple alternative signaling pathways that could be targeted to address pathological angiogenesis in breast cancer. Approaches targeting the pathways detailed below have been summarized in **Table 2** and offer the potential to improve upon current therapeutic strategies by addressing resistance mechanisms and promoting the efficient long-term stabilization of tumor blood vessels.

**Table 2:** Summary of Therapies Targeting Novel Tumor Angiogenesis Pathways in Breast Cancer.

Therapies	Mechanism of Action	Associated Trials	Outcomes
BMP9			
Recombinant BMP9	Signals through receptor complex to induce downstream signaling.	Pre-Clinical	Reduces tumor growth and vascularization in a mouse model of glioblastoma (119).
PF-03446962	Monoclonal blocking antibody for ALK1 that prevents the binding of BMP9 to endothelial cells.	Pre-Clinical	PF-03446962 as a monotherapy for breast cancer demonstrated no significant anti-tumor effects (120). A greater reduction in tumor growth was observed when given in combination with either Bevacizumab or a VEGFR tyrosine kinase inhibitor (120).
Dalantercept	Alk1-Fc ligand trap that sequesters Alk1 ligands to prevent receptor activation.	Phase-1 Clinical	Inhibits tumor angiogenesis as a monotherapy, or combination therapy (121).
		Phase-2 Clinical	Insufficient single agent activity to justify further investigation (122). Discontinued as a combination therapy with Axitinib due to an overall lack of efficacy (123). Side-effects include peripheral edema, nosebleeds and telangiectasias (121, 124, 125).
Shh			
Pristimerin	Inactivates Shh/Gli1 and related downstream signaling.	Pre-Clinical	Inhibition of Shh-mediated endothelial proliferation, migration, invasion and sprouting during the early stages of angiogenesis *via* VEGF dependent mechanisms (126–128). Blocks the recruitment of pericytes that stabilize newly formed vessels at later stages (128).
Cox-2			
Celecoxib	Improves efficacy of anti-angiogenic therapies through an anti-VEGF pathway.	Pre-Clinical	*In vivo* models have shown a reduction in tumor growth and metastases (129, 130) Associated with an increased risk of CV toxicity, GI complications, and death (131).
		Multiple Clinical Trials
P38-MAPK			
Ralimetinib	P38-MAPK selective kinase inhibitor.	Pre-Clinical	Reduced breast cancer cell invasiveness *in vivo* (132).
		Phase 1	Ralimetinib in combination with Tamoxifen showed modest improvements in progression free survival in AMBC and is an acceptable, safe, and tolerable therapy (133, 134).
		Phase 2	Trial was terminated due to lack of participant enrollment (Identifier NCT02322853).

### Bone Morphogenetic Protein-9 (BMP9) Signaling

**Figure 2:**
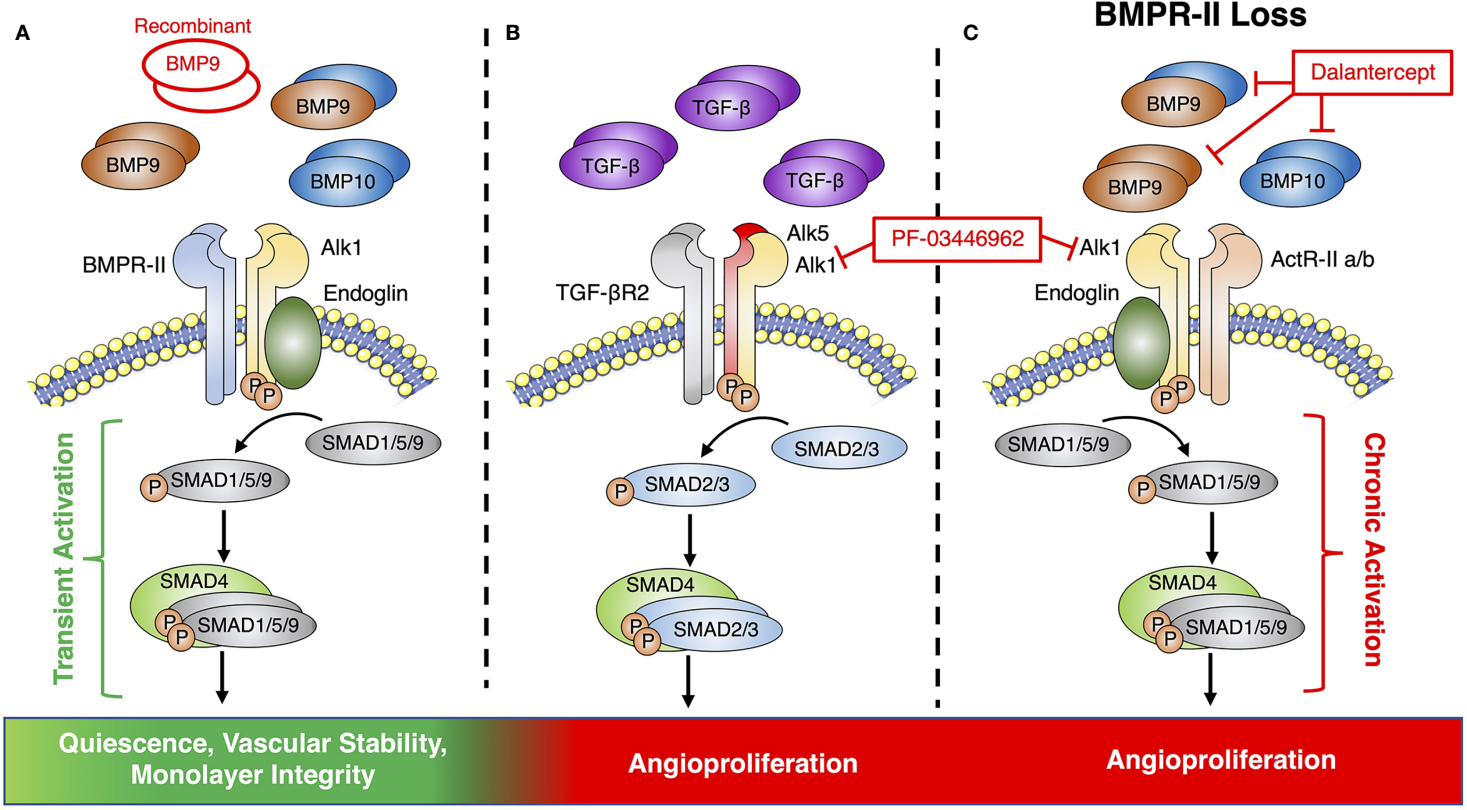
Endothelial BMP9 and TGFβ signaling *via* the Alk1 signaling axis. **(A)** Conventional endothelial BMP9 and BMP10 signaling *via* a receptor complex of Alk1 and BMPR-II leads to transient activation of downstream SMAD signaling, endothelial quiescence and vascular stability. Previous therapeutic studies have pursued vascular normalization by supplementing this pathway with recombinant BMP9. **(B)** Alk1 can also partner with ALK5 and TGFβR2 to form a receptor complex for TGFβ to promote angiogenesis. **(C)** BMPR-II loss causes signaling *via* alternative type-II receptors, such as ActR-IIa/b, which can promote angiogenesis *via* the chronic activation of canonical SMAD signaling. Therapeutic targeting of pre-angiogenic signaling of TGFβ or BMP9 *via* Alk1 has been pursued by receptor blockade with PF-03446962 or the Alk1-Fc ligand trap, Dalantercept.

Since its discovery as a ligand for the orphan receptor, Activin receptor-like kinase 1 (Alk1), BMP9 has received significant attention as a mediator of vascular growth, stability, and integrity (135–137). BMP9 is a member of the transforming growth factor-β (TGFβ) superfamily and signals through complexes of type-I and type-II receptors (138). Alk1 is the dominant type-I BMP receptor in the endothelium and can pair with either BMPR-II or the activin type-II receptors (ActR-IIa and ActR-IIb) to form a receptor complex for BMP9, BMP10 and heterodimers of the two ligands, which are present at active concentrations in the circulation (138, 139) (**Figure 2A**). Receptor binding can induce both canonical signaling through phosphorylation of the SMAD 1, 5 and 9 transcriptional mediators, as well as non-canonical signaling *via* a variety of pathways, including Notch, Wnt, and p38 MAPK (140–143). Although recent studies have shown that the majority of BMP9 in the circulation exists as a heterodimer with a BMP10 subunit (139), most investigations into the potential of BMP9 to influence angiogenesis have explored the angiogenic potential of BMP9 homodimers alone.

BMP9 was originally shown to serve as a vascular stability factor that suppresses endothelial proliferation *in vitro* (144, 145) and angiogenesis *in vivo* (136). In cancer, multiple studies have shown that BMP9 can mediate the maturation phase of angiogenesis and may contribute to blood vessel normalization through the inhibition of endothelial proliferation and migration, as well as the recruitment of pericytes (142, 146–148). Deletion of *Gdf2*, the gene encoding BMP9, leads to enhanced tumor growth and increased lung metastases in the syngeneic orthotopic E0771 mouse model of metastatic breast cancer (149). This finding was associated with increased tumor vascular density and reduced mural cell coverage (149). The administration of recombinant BMP9 has also been shown to reduce tumor growth and vascularization in mouse models of glioblastoma (119). This approach may be a promising option for breast cancer, as it could be given in conjunction with current chemotherapies.

While there is substantial evidence supporting this anti-angiogenic and vascular stabilizing role for BMP9 in cancer, these effects appear to be highly context dependent (150–155). Multiple *in vivo* studies have also reported a pro-angiogenic role for BMP9 (151, 153) in the tumor microenvironment that is mediated *via* a synergistic activity with TGFβ (156, 157). Interestingly, TGFβ has been shown to signal *via* Alk1 in some endothelial cell models through a complex containing the type-II TGFβ receptor and Alk5, which is known as the conventional type-I receptor for TGFβ (158) (**Figure 2B**).

PF-03446962 is a monoclonal blocking antibody for Alk1 that was developed to target the pro-angiogenic effects of TGFβ, but has also been shown to prevent the binding of BMP9 to endothelial cells (159). Although pre-clinical studies using PF-03446962 as a monotherapy for breast cancer demonstrated no significant anti-tumor effects, a greater reduction in tumor growth was observed when it was given in combination with either Bevacizumab or a VEGFR tyrosine kinase inhibitor (120). An additional anti-angiogenic therapy targeting the BMP9-Alk1 axis is Dalantercept, an Alk1-Fc ligand trap that sequesters Alk1 ligands and prevents them from activating the receptor (121). Unlike PF-03446962, Dalantercept was shown to inhibit tumor angiogenesis as either a monotherapy or in combination with other treatments (121). While Dalantercept demonstrated promising efficacy and was well-tolerated in early studies (121), later clinical trials demonstrated no benefit in endometrial cancer (122) and it has since been discontinued as a combination therapy for advanced clear cell renal cell carcinoma due to an overall lack of efficacy (123, 160). Overall, these findings call into question the ultimate merits of blocking signaling *via* the BMP9-Alk1 axis as a viable clinical approach for breast cancer.

In addition to the contradictory information surrounding the pro- and anti-angiogenic effects of BMP9 signaling, the therapeutic potential of targeting this pathway is further complicated by the fact that BMP9 does not act on the endothelium in isolation, but can also influence the growth of tumor cells, as well as interactions between the two cell types in the tumor microenvironment (161, 162). A study by Eleftheriou and colleagues indicated that inhibiting BMP9 decreases tumor volume, while increasing vascular branching and metastases (154), suggesting that BMP9 may cause certain cancer cells to proliferate, while also promoting vascular quiescence. Along this line, a pro-proliferative effect of BMP9 has been reported in ovarian, liver, bladder, and pancreatic cancer cell lines, but not in breast cancer (151, 153–155).

Beyond its role in cancer, the angiogenic actions of BMP9 have also been explored in other pathologies, including pulmonary arterial hypertension (PAH), a disease of occlusive pulmonary vascular remodeling that is linked to excessive endothelial cell proliferation and loss-of-function mutations in the gene encoding BMPR-II (163). This work may help to explain the contradictory actions of BMP9 in cancer by identifying BMP9 signaling as an “angiogenic switch” that can either promote or prevent angiogenesis, based on the availability of BMPR-II as its type-II receptor (164). Under this model, the loss of BMPR-II in the endothelium drives BMP9 signaling *via* alternative type-II receptors, such as ActR-IIa or ActR-IIb, resulting in prolonged activation of the canonical signaling pathway and a cancer-like shift of the BMP9 response towards pathological endothelial proliferation (**Figure 2C**).

Although a similar shift has not yet been identified in cancer, the recognition of a central role for type-II receptor utilization in regulating the balance between the pro- and anti-angiogenic effects of BMP9 highlights the value of a more nuanced understanding of BMP9 signaling in disease. Such an approach could improve on the poor efficacy of ligand traps like Dalantercept and blocking antibodies like PF-03446962, which block all BMP9-related signaling, including the beneficial effects of BMP9 on vascular growth and stability *via* BMPR-II. Blockade of the vascular stabilizing effects of BMP9 could also explain why Dalantercept trials demonstrated poor efficacy in human cancers, accompanied by side-effects linked to vascular instability, such as peripheral edema, nosebleeds and telangiectasias (121, 124, 125). Profiling the relative expression of type-II BMP receptors in the tumor endothelium could also help to identify breast cancer tumors that would benefit from recombinant BMP9 therapy, versus those for which this treatment may enhance disordered vascularization.

### Shh Signaling

**Figure 3:**
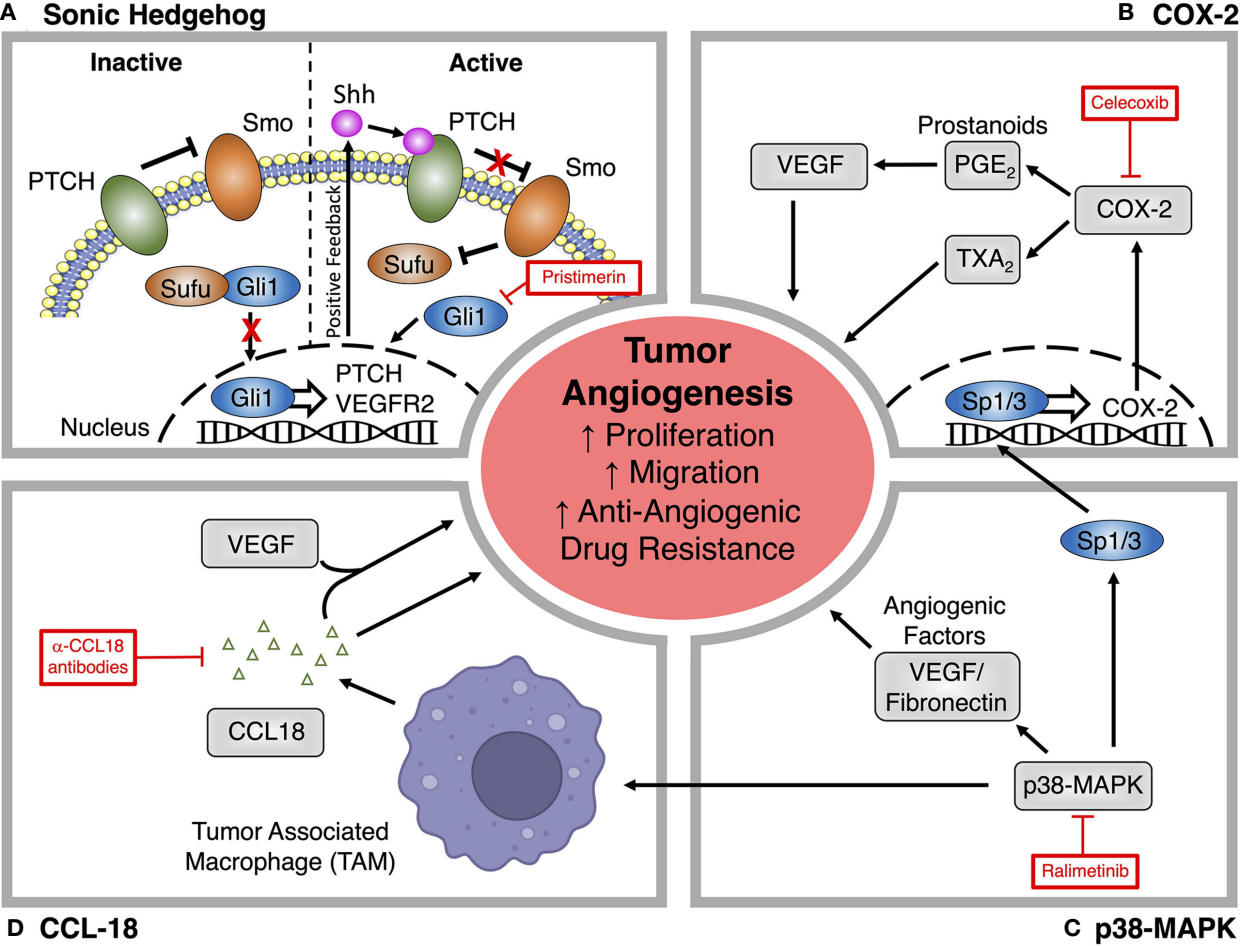
Alternative signaling pathways for targeting tumor angiogenesis. **(A)** Pristimerin inhibits the pro-angiogenic actions of Sonic hedgehog (Shh) signaling by targeting downstream effects *via* the transcription factor Gli1. **(B)** The COX-2 inhibitor Celecoxib prevents the synthesis of downstream prostanoids, which can promote tumor angiogenesis, endothelial proliferation and migration through the production of angiogenic factors like VEGF. **(C)** Ralimetinib blocks p38-MAPK signaling, which promotes the deposition of angiogenic factors within the tumor microenvironment and can also promote angiogenesis through actions on tumor associated macrophages (TAM) and COX-2 expression. **(D)** Production of CCL18 by TAMs promotes angiogenesis through VEGF-dependent and independent mechanisms. CCL18 neutralizing antibodies have been explored as a novel treatment for tumor angiogenesis.

Recently, the Shh pathway has emerged as a main regulator of tumor angiogenesis and a promising therapeutic target (165). There are three different pathways involved in Hedgehog signaling, Shh, Indian-Hedgehog, and Desert-Hedgehog (166). The Shh pathway signals through active and inactive pathways (**Figure 3A**). Inactive signaling occurs due to the ligand independent interaction of Shh and the transmembrane protein Patched (Ptch1), whereas active signaling occurs through factors downstream of smoothened (Smo), a G protein-coupled receptor protein that is required to activate glioma associated oncogenic (Gli) proteins (167, 168) (**Figure 3A**). The active Shh signaling pathway is induced when Shh binds and inactivates Ptch1 (169). This binding stimulates the release of Smo, which activates the transcription factor Gli homolog 1 (Gli1) and allows for the regulation of genes involved in cell growth, differentiation, drug resistance, and angiogenesis (167, 169).

Shh signaling can promote vascularization through its actions on both the VEGF/VEGFR2, and Ang/Tie2 signaling pathways to promote endothelial proliferation, migration, invasion, and new vessel maturation (170, 171). As the main transcription factor of the Shh pathway, Gli1 upregulates the release of multiple pro-angiogenic molecules, including VEGF and the VEGFR1 and VEGFR2 co-receptors cysteine-rich angiogenic inducer 61 (CYR61) and neuropilin 2 (NRP2) (172–175). Shh signaling can also facilitate tumor vascularization by upregulating the expression of VEGFR2 on the surface of cancer cells (165). Clinically, dysregulation of Shh signaling plays a key role in breast cancer progression and metastasis and is a measure of poor prognosis (176–178). Overexpression of Gli1 is also correlated with increased vascular density in breast cancer (165). There are numerous potential therapeutic targets in the Shh pathway that could influence tumor angiogenesis.

Pristimerin is an anti-inflammatory quinonemethide triterpenoid compound that is being explored as a potential anti-angiogenic candidate targeting Shh signaling (126). Pristimerin exerts its anti-angiogenic effects by inactivating Shh/Gli1 and related downstream signaling, resulting in the inhibition of Shh-mediated endothelial proliferation, migration, invasion and sprouting during the early stages of angiogenesis *via* VEGF dependent mechanisms (126–128). Pristimerin can also act at later stages to block the recruitment of pericytes that stabilize newly formed vessels (126–128). In addition to the effects of Pristimerin on angiogenesis, it can induce cancer stem cell toxicity in breast cancer (179), indicating a potential to impact the vascular mimicry-mediated resistance that limits other anti-angiogenic approaches. Overall, it appears that Pristimerin is efficacious as an anti-angiogenic therapy in cancer. However, these findings have not yet been translated into clinical success and the precise mechanisms driving these effects remain unclear (180, 181).

### COX-2 Inhibition

COX is the rate-limiting enzyme for the synthesis of prostaglandins, with COX-2 being the dominant isoform driving prostaglandin production during inflammation (182). It has been previously confirmed that the expression of COX-2 in the endothelium can be induced by hypoxia-stimulated release of VEGF through a mechanism that is dependent on the activation of the p38-MAPK and c-Jun N-terminal kinase (JNK) pathways (183). P38-MAPK signaling can also upregulate COX-2 expression within tumors *via* the Sp1/Sp3 transcription factors (184). The prostaglandins produced by COX-2 stimulate the production of angiogenic mediators, like VEGF, in cancer and endothelial cells (**Figure 3B**), driving a positive feedback loop between the two cell types (185). The expression of COX-2 is 90% elevated in colorectal cancer, 70% in lung cancer, and 37% in breast cancer and has been correlated with a poor prognostic outcome (186).

A study by Xu et al., has predicted that COX-2 inhibitors targeting tumor inflammation and angiogenesis could enhance the activity of conventional anti-angiogenic therapeutics against pre-established metastases and improve the prognosis for patients with COX-2 overexpressing tumors (185). Celecoxib is a COX-2 inhibitor that has been used to treat angiogenesis in breast cancer (129, 130). Although Celecoxib may improve the efficacy of anti-angiogenic therapies through a distinct anti-VEGF pathway, this approach is also limited by dose-dependent side effects that have reduced enthusiasm for this approach (129, 130). In clinical trials, patients treated with COX-2 inhibitors have an increased risk of CV toxicity, GI complications, and death (131, 187, 188). Whilst initial results are promising, additional controlled clinical trials are needed to confirm how COX-2 inhibitors perform when combined with VEGF inhibitors for the treatment of AMBC (128, 185). Due to the abundance of clinically available COX-2 inhibitors and years of experience with these drugs in various patient populations, a demonstration of clinical efficacy from these trials could allow for the rapid incorporation of COX-2 inhibitors into conventional therapeutic regimens.

### P38-MAPK Pathway

P38-MAPK is a signal transduction mediator that is involved in inflammation, the cell cycle, cell death, cellular development, differentiation, senescence and tumor development (189). A meta-analysis by Limoge et al. indicated that p38 target genes and p38-MAPK signaling are elevated in breast cancer, and have been linked to increased tumorigenesis, invasiveness, metastasis, disease recurrence, and poor outcomes (190). P38-MAPK also contributes to the formation of blood vessels in tumors by enhancing the production, and deposition of pro-angiogenic factors that alter the tumor microenvironment (**Figure 3C**), including pro-angiogenic cytokines and fibronectin, a component of the extracellular matrix that serves as an anchor for VEGF (190).

The p38-MAPK pathway may also contribute to angiogenesis through its effects on TAMs and neutrophils, central immune components of the tumor microenvironment. TAMs contribute to vascular sprouting by bridging tip cells, as well as by secreting a variety of angiogenic growth factors such as TGFβ, VEGF, endothelial growth factor, and a variety of chemokines (34, 191–193). The p38-MAPK pathway is also known to regulate TGFα, TGFβ, and interleukins, which are all established drivers of TAM function and play a role in the colonization of cancer cells and angiogenesis (194). In addition, p38 may also modulate VEGF-mediated endothelial migration, further contributing to angiogenesis (195).

Genetic inactivation of p38 has been shown to reduce tumor angiogenesis (132, 190), offering promise that anti-p38 drugs could be a new therapeutic option for treating AMBC, as well as breast cancer in general. The pharmacological inhibition of p38 significantly reduces tumor growth, angiogenesis, and lung metastasis (196). P38 inhibition can also help to improve the efficacy of current anti-angiogenic therapies, as TAMs have been shown to contribute to the resistance of VEGF-targeted anti-angiogenics (197). Ralimetinib, a p38-MAPK selective kinase inhibitor, has been shown to reduce breast cancer cell invasiveness *in vivo* (132). A phase 1 clinical trial assessing Ralimetinib in combination with Tamoxifen demonstrated that it could provide modest improvements in progression free survival in AMBC and is an acceptable, safe, and tolerable therapy (133, 134). A phase 2 clinical trial of Ralimetinib was initiated to address the efficacy and safety of p38-MAPK inhibition in AMBC; however, the trial was terminated due to lack of participant enrollment (identifier NCT02322853). Despite the early promising findings, a major concern associated with this therapeutic approach is the fact that p38 is a well-recognized tumor suppressor (198), suggesting the need to balance any beneficial effects on tumor angiogenesis against potentially damaging actions on the tumor itself. The time of administration is also an important factor in this therapeutic approach, as p38 inhibition is impacted by circadian rhythms (199).

### CCL18 in Tumor Angiogenesis

Several chemokines are currently being investigated for their association with angiogenesis However, CCL18 appears to be the most promising. CCL18 is the most abundant and specific chemokine released from TAMs, and serves the function of recruiting B-cells, T-cells, and natural killer (NK) cells (200). It is also essential for the promotion of tumor angiogenesis and endothelial cell survival (201) and is linked to poor prognosis in breast cancer (200). CCL18 can either work synergistically with VEGF, or through VEGF-independent mechanisms (**Figure 3D**), to promote migratory and angiogenic effects both *in vitro* and *in vivo (* 202). Beyond its pro-angiogenic activity, CCL18 can also promote endothelial to mesenchymal transition (EndMT) in the tumor microvasculature, which can lead to loss of cell-to-cell junctions, and enhanced invasion and migration (202).

Therapeutically, targeting CCL18 represents a promising treatment for patients with resistance to anti-VEGF therapies. Neutralizing antibodies towards CCL18 do not only block its effects on tumor angiogenesis though VEGF-independent mechanisms, but may also improve the response to other anti-angiogenic therapies *via* TAM-driven processes (202). CCL18 should be further assessed for biological activities using mechanistic studies and the pre-clinical and clinical evaluation of novel inhibitors (203).

In conclusion, drug resistance, severe side-effects, limited survival advantage, and enhanced cancer regrowth rates associated with current anti-angiogenic therapies highlight the critical need for novel targets and therapies targeting this aspect of tumor growth and metastasis. Beyond the novel therapies discussed in this review, non-coding RNAs, including long non-coding and circular RNAs are being investigated as potential master regulators of tumor angiogenesis. These therapies are advantageous as they have multiple targets and broad affects are difficult to achieve through approaches targeting individual pathways or ligands. Increased understanding of the factors that stimulate tumor angiogenesis and the interactions between these novel pathways and more established mediators may also allow for a refinement of therapeutic approaches, without the need for new drugs. Together these advances offer the promise of new treatment options that will improve the prognosis for AMBC patients.

## Author Contributions

JH contributed to the conception and design of the review article. JH wrote each section of the manuscript and prepared the figures. MO provided feedback on the conception and contributed to the revision of the manuscript and figures. All authors contributed to the article and approved the submitted version.

## Funding

This work is funded by the Canadian Institutes of Health Research. Grant #: PJT-178078.

## Conflict of Interest

The authors declare that the research was conducted in the absence of any commercial or financial relationships that could be construed as a potential conflict of interest.

## Publisher’s Note

All claims expressed in this article are solely those of the authors and do not necessarily represent those of their affiliated organizations, or those of the publisher, the editors and the reviewers. Any product that may be evaluated in this article, or claim that may be made by its manufacturer, is not guaranteed or endorsed by the publisher.
